# Supporting the victims of domestic violence in Iran: two decades of effort

**DOI:** 10.5249/jivr.v13i2.1638

**Published:** 2021-07

**Authors:** Faraneh Ghaffarihosseini, Amir Hossein Jalali Nadoushan, Kaveh Alavi, Jafar Bolhari

**Affiliations:** ^*a*^ Iran University of Medical Sciences, Tehran, Iran.; ^*b*^ Mental Health Research Center, Community Mental Health Research, Iran University of Medical Sciences, Tehran, Iran.; ^*c*^ Mental Health Research Center, Iran University of Medical Sciences, Tehran, Iran.; ^*d*^ Spiritual Health Research Center, Iran University of Medical Sciences, Tehran, Iran.

**Keywords:** Domestic violence, Partner abuse, Child abuse

## Abstract

For years there were no organized supporting system helping victims of domestic violence in Iran. 16 years ago Ministry of Interior started a national survey which led to try legislating bills in order to improve preventive and supporting services. This has inspired many health care professionals, including Ministry of Health, Treatment and Medical Training, to pursue this field for research. Since then, many studies has been done; which were reviewed in this paper. We tried to build a stepping stone for the future researchers and activists, since despite all what has been done, still there is no legislated bill or enough organizations to protect the victims.

## Brief introduction of what has been done

Domestic violence is defined by the World Health Organization (WHO) as any behavior which leads to physical, psychological or sexual harm within an intimate relationship.^1^ That also includes various controlling behaviors, for instance isolating one from her/his family and friends and deprivation of basic needs. The WHO estimated that 15-71% of women had been physically or sexually assaulted by their partners.^1^ Men are also victimized, but much less than women, thus research is less common,^2^ although this can be due to less reports of domestic violence by men in general. Despite the particular attention to women's constructive role, in strengthening family and other social scenes, there is no organized national policy and program supporting them against domestic violence.^3^ The first national survey took place in year 2004, estimating the rate of domestic violence against women (including psychological, verbal, physical, sexual and financial abuse). It was later categorized geographically. Based on which Iranian women are victimized the most, during the first year of marriage, financial difficulties, after giving birth, midlife and pregnancy. These women considered their husbands' characteristics as the main cause of conflicts turning to domestic violence, and after that, they found their husbands' family and their own families were to be blamed. But it should be noted that they also blamed themselves as much as they blamed their husbands.^4^

This study has been followed by many researches, which in 2012^5^ led to a policy paper planning to pursue three goals: 1. Reducing new and further domestic violence against victims. 2. Increasing literacy of different groups in the field of domestic violence prevention and control. 3. Increased counseling and social support for women, children and the elderly in defined facilities. Based on these policies' pilots for more active advocacy, Primary Health Care (PHC) oriented and supporting programs had been designed, which were found promising.^6,7^

Two qualitative and quantitative researches have been executed in five provinces to investigate potential risk factors for domestic violence in Iran. Four of the most violent provinces and the one with the least domestic violence prevalence reported was selected. It showed that the frequency of exposure to violent behavior of a woman or a man from her/his spouse is respectively 53/7% and 40/4%, which was twelve percent less than the statistics showed before.^4^ Other studies which precede domestic violence, were more limited and had fewer sample size. A meta-analysis based on the 31 articles from 2000 to 2014, with a representation of a 15,514 sample size estimated the prevalence of domestic violence about 66%^8^ which is closer to the national survey.^4^ Most recent related article which had been published titled "Domestic Violence against women in Shiraz Southwestern Iran", showed that more than 50% of married women in southwestern Iran were being somehow abused by their partners.^9^

## Results originated from the projects

According to these studies there are plenty reasons which lead to domestic violence. Among them, are general reasons such as partner's drug addiction^5,10-12^ or his criminal record^11,12^ and women being younger of age or being newlyweds.^4,5,13^ There were also controversial results, for instance one cross-sectional study showed that weak religious beliefs in men lead to more violence^12^ as opposed to another cross sectional one, which found religious husbands more abusive.^11^ Another example would be education. One study stated that higher literacy, not only negatively correlated with the prevalence of domestic violence^5^ but also encouraged women to seek justice.^14^ Also whether it is due to inability to bear a child or simply the pregnancy, women suffer a lot from physical, verbal or sexual abuse.^4,5,15,16^ Working women did not have a less violent relationship in all the cases.^5^ But the two most important correlations, which were found repeatedly were financial problems. The other one being women witnessing their violence against their mothers during their childhood.^5,9,17^ Having a mother whom was abused also stated to have effect on small boys, which will guide them, later in life, to abusive behavior towards their partner.^9,14^

Based on these studies variant pilots have been planned. One of the proceedings of the mental health office is implementing a pilot project to prevent child abuse in one hospital (Mohammadi Hospital) in southern Iran. This center has a specialized unit that provide services to children and families whom somehow were abused.^18^

While asking about the definition of domestic violence, men mostly complained about psychological and verbal violence. On the other hand, women complained about physical violence the most. Men noted violence more reasonable in some circumstances, one being woman's refusal of having intercourse. This was followed by women having more tolerance towards violence. In other words, women have learnt that they deserve to be beaten. It also seems that general violence against children is more acceptable in various places and even has been considered useful.^19^

Much like the results of quantitative part, financial problem plays an important part in the minds of both gender.^17,9,5^

Ministry of Health recommends to many organizations establishing central national committee for safety and prevention of domestic violence; developing/promoting domestic violence program in Ministry of Health and Medical Education, judiciary, legislative members, mass media and other stakeholders in the program.^5^ One of the main efforts in this field is training programs on domestic violence prevention for couples before marriage: increasing knowledge, changing attitude toward domestic violence and training social skills like anger/stress management, problem-solving in general population (men, women) are recommended.^19^

## Conclusion

After all, there are no laws against domestic violence, despite all the damage it costs. All these efforts have come to an unlegislated bill which has been reduced to a financial penalty. There is no day dedicated to the victims of domestic violence, nor to conducting a national survey. We believe that since domestic violence is an issue with more than just health and medical aspects, only by analyzing it from other perspective, such as sociological, financial and ideological, as well, we, as a nation, would find a way to help those in need. 

**Special area for attention**

Empowering programs for families of the disabled and the elderly against domestic violence leads to a better quality of life in victims. It was seen that the families with disabled and elderly members, are significantly exposed to domestic violence more than others. Thus, these families should be particularly contributed encourage to join empowering programs for domestic violence control and special training programs. Necessary legal context for interventions, such as providing a multi-service, interdisciplinary programs (including health care centers, day clinics, adult day care centers and home visits), which supply services for the elderly and their care givers, would reduce the burden of caring for them and therefore could decrease the rate of their abuse. In addition educational courses of geriatric medicine would help.^19^

Although, to this day, many plans and pilots have been run, a valuable improvement is only achieved when all the different parts of government, religious authorities, NGOs, and collaboration with regional and international agencies in Iran, work together.

**Acknowledgements**

Qualitative and quantitative data presented from the policy paper, are part of a project designed by the Ministry of Health and Medical Education of Iran, with the support of WHO. We are grateful to all researchers and patients who had been cooperative.
